# Doctoring from home: Physicians’ perspectives on the advantages of remote care delivery during the COVID-19 pandemic

**DOI:** 10.1371/journal.pone.0269264

**Published:** 2022-06-02

**Authors:** Matthew J. DePuccio, Alice A. Gaughan, Karen Shiu-Yee, Ann Scheck McAlearney

**Affiliations:** 1 Department of Health Systems Management, College of Health Sciences, Rush University, Chicago, Illinois, United States of America; 2 Center for the Advancement of Team Science, Analytics, and Systems Thinking in Health Services and Implementation Science Research (CATALYST), College of Medicine, The Ohio State University, Columbus, Ohio, United States of America; 3 Department of Family and Community Medicine, College of Medicine, The Ohio State University, Columbus, Ohio, United States of America; 4 Department of Biomedical Informatics, College of Medicine, The Ohio State University, Columbus, Ohio, United States of America; University of Oklahoma Health Sciences Center, UNITED STATES

## Abstract

**Background:**

During the COVID-19 pandemic, stay-at-home orders as well as shortages of personal protective equipment forced primary care physicians (PCPs) to transition rapidly from in-person visits to telehealth. While telehealth expanded extensively in a short period of time, research about the consequences of the shift to remote care is lacking. The objective of this qualitative study was to examine how telehealth benefited PCPs and their patients during the COVID-19 pandemic.

**Methods:**

From July to August 2020, semi-structured interviews were conducted with 20 PCPs associated with a single academic medical center to examine their perspectives about delivering care remotely during the COVID-19 pandemic. All interviews were recorded, transcribed verbatim, coded, and analyzed using deductive thematic analysis.

**Results:**

PCPs identified several benefits of remote care delivery for both physicians and patients. They indicated that (1) patients were reassured that they could receive safe and timely care, (2) remote visits were convenient for patients, (3) patients were comfortable receiving care at home, and (4) video visits enhanced patient- and family-centered care during the COVID-19 pandemic. Participants also noted that (1) telehealth accommodated working from home, (2) physicians were equitably reimbursed for telehealth visits, and that (3) telehealth promoted physician work-life balance.

**Conclusions:**

Our data provides preliminary evidence that PCPs and their patients had positive experiences with remote care during the early months of the COVID-19 pandemic. Physicians identified opportunities by which telehealth could enhance the delivery of patient-centered care by allowing them to see patients’ home environments and to engage family members and caregivers during telehealth visits. More research is needed to understand how to sustain these benefits beyond the global COVID-19 pandemic and ensure patients’ needs are met.

## Introduction

In spring of 2020, healthcare practices across the U.S. rapidly implemented telehealth to expand remote care options in response to the emergence of coronavirus disease 2019 (COVID-19) [1–3]. Telehealth refers to a variety of synchronous (telephone or video) and asynchronous (patient portals or online medical records) activities that allow physicians to provide patient care at a distance [2]. Experts expect remote care options to remain a central component of healthcare delivery beyond the COVID-19 pandemic because of its potential to effectively address a wide range of patient needs; for example, chronic disease management, mental health follow-ups, and counseling services [1].

Synchronous video calls between physicians and patients (i.e., video visits) are a specific type of telehealth that continues to proliferate as the COVID-19 pandemic continues [2, 4, 5]. Previous research has demonstrated that video visits offer certain benefits to patients including staying connected to their physicians, avoiding crowded waiting rooms [6], and saving time by not having to travel long distances for in-person visits [7, 8]. Furthermore, one study demonstrated that patient satisfaction scores were higher for patients who had a video visit versus an in-person visit and who received care during the COVID-19 pandemic versus before the COVID-19 pandemic [9]. In practice, physicians can use telehealth, and video visits specifically, to remotely monitor and manage patients’ acute and chronic medical needs and triage patients who may potentially need more intensive treatment [10]. Given such benefits, researchers and practitioners continue to promote the ongoing use of video visits as a remote care option [11].

As we have begun to understand the short- and long-term implications of this dramatic shift towards telehealth [12], as well as the barriers and facilitators to its use during the COVID-19 pandemic [13, 14], we still do not have a clear understanding of how telehealth, as a type of care delivery model, has benefited physicians and patients, especially beyond increased convenience [15], and improved satisfaction [16]. Further, as the use of telehealth proliferated, physician concerns about the adequacy of remote physical examinations [7, 17], which could result in unnecessary referrals and additional testing [18], raise questions about how physicians experienced telehealth in the context of delivering fewer in-person visits during the COVID-19 pandemic [19], and how these experiences may have impacted patients. Answering these questions can help illuminate the aspects of telehealth that contribute to high-quality, safe, and affordable patient care—priorities that continue to be the focus of policymakers and healthcare institutions [20]. Furthermore, as healthcare practices continue to invest in building up their telehealth infrastructure, improved understanding of physicians’ perceptions about the benefits of this care modality can help identify opportunities to sustain its use and to better address patients’ needs in the future. Thus, the objective of our study was to explore the perspectives of primary care physicians (PCPs) who used telehealth during the early months of the COVID-19 pandemic to better understand how this remote care option benefited them and their patients.

## Materials and methods

### Study setting

At the end of 2019, the Division of General Internal Medicine and the Department of Family and Community Medicine at a Midwestern academic medical center set the goal of transitioning at least 30% of routine, in-person primary care services to telehealth (i.e., telephone and video) over the next 3–5 years. A telehealth workgroup was formed to identify barriers to implementation and was focused on physician telehealth training and securing buy-in from clinicians and leadership at the institution. Although patients expressed a willingness to engage in telehealth, the workgroup discovered that patients had many questions around the technical requirements and preparations to have a video visit and how such visits would be covered by their insurance. In March 2020, the medical center responded to the emergence of COVID-19 by converting in-person, non-emergent primary care visits to telehealth across the service area. The percentage of video and telephone visits increased from 1.2% to 91.8% between the first and last weeks of March [21]. At one point, 98% of the 1,800 physicians across the medical center were conducting about 2,500 telehealth visits per day [21].

### Study design and population

We designed a qualitative study including video and phone interviews with PCPs to learn how physicians were impacted by the rapid expanded use of telehealth in response to the emergence of COVID-19. Primary care physicians from General Internal Medicine and Family and Community Medicine responded directly to requests to participate in our study. Physicians were interviewed between July and August 2020 and were asked about their experience with the expanded use of telehealth and how they were affected by the transition from in-person visits to telehealth. Specifically, as part of the larger study [14], we asked physicians to describe patient-related challenges associated with telehealth use during the pandemic and how the shift to telehealth changed the nature of their work with support staff (see S1 File). However, for this analysis, we were specifically interested in PCPs’ perspectives about the potential benefits of delivering primary care via telehealth, to better understand what under what circumstances and for which conditions physicians may be receptive to using telehealth as a replacement for in-person care [16], as well as what aspects of telehealth were advantageous, particularly during the COVID-19 pandemic.

### Interview procedures, data collection and analysis

Interviews were conducted using a semi-structured interview guide (see S1 File) that asked open-ended questions about the early impacts of COVID-19 on the work of PCPs. Questions were developed using the extant COVID-19 literature with input from a panel of health services researchers and a PCP. We piloted our guide with several PCPs prior to recruitment to ensure our questions were clear and allowed PCPs to freely express their perspectives and ideas. Interviews lasted approximately 35 minutes each and all interviewees provided informed consent to participate in the study. All interviews were audio-recorded, transcribed verbatim, and de-identified. The institutional review board of The Ohio State University approved this study.

Interview transcripts were analyzed using deductive dominant thematic analysis [22, 23], allowing for categorization of data based on general themes derived from the interview guide, as well as identification of emergent themes. This approach allowed for comparison of themes across interviews and enabled us to characterize advantages associated with remote delivery of primary care. Specifically, two members of the research team reviewed two of the interview transcripts and developed a preliminary coding dictionary based on the questions in the semi-structured interview guide while also allowing for additional codes to emerge based on topics discussed during the interviews. The codebook was refined, and one member of the research team applied the finalized codes to remaining transcripts. The research team met regularly to ensure coding consistency across the interviews and to resolve coding discrepancies. We used the ATLAS.ti (version 8.4.4) qualitative data analysis software to support our coding and analysis process.

## Results

### Characteristics of study participants

The interviewees included 20 PCPs who had, on average, 16 years of experience practicing primary care with a range between 3 to 41 years. Fifty percent of interviewees reported practicing medicine between 0–15 years while 50% reported practicing medicine for more than 15 years. Most interviewees were female (55%) and Family and Community Medicine physicians (65%).

### Physicians’ perspectives on the advantages of telehealth for patients

Interviewees acknowledged that video visits were beneficial in four main ways: (1) patients were reassured that they could receive safe and timely care, (2) remote visits were convenient for patients, (3) patients were comfortable receiving care at home, and (4) video visits enhanced patient- and family-centered care during the COVID-19 pandemic. We provide greater detail about these themes in the following sub-sections, including quotations that support the characterization of these themes. Additional example verbatim quotations are presented in Table 1.

**Table 1:** Physicians’ perspectives about advantages of providing remote care for patients.

Themes	Verbatim comments from primary care physicians
**A reassuring alternative to in-person care**	*[Telehealth] has given us the chance to prevent*, *well decrease*, *the risk of exposure not just from COVID but in the future…from flu as well*.
	*We are looking at some of the in-person visits that are scheduled and saying*, *‘Hey*, *this person is scheduled for an anxiety follow-up and if they’re okay with it*, *we can switch to telehealth*.*’ Just again*, *[to] minimize people in the office*.
**Convenience of video visits**	*For my other patients who may have transportation issues*, *I don’t have to worry*, *because [there’s] nothing worse than patients calling you*, *very upset*, *‘The transportation didn’t come pick me up today’…that’s horrible*.
	*I think that for the patients who engage in telehealth*, *I think the feedback has been generally positive around*, *‘I’m so glad I could get in this way*. *This is so convenient*.*’ I’m glad that I could get a doctor’s appointment and not have to leave work*.*’*
**Comfort of receiving care at home**	*I do more counseling*, *much more counseling for patients through this kind of interaction because they are really in their comfort zone…They allow me to talk about weight and nutrition and I’m like*, *‘Show me that bottle that you’re drinking from*.*’*
	*One of my patients had a stroke during the pandemic*, *and can’t drive*, *and feels pretty bad*. *But I was still able to do a video visit with her and her family…And maybe that was something that we couldn’t have done before*.
**Enhanced patient- and family-centered care**	*Their wife walks by and says*, *‘You better remember to tell him about your left ankle that’s been hurting you*.*’ So*, *it’s just a different way of providing care*, *and I think for the better*.
	*The other one is you also get the perspective of family members*. *Family members can be present…And you almost get the global understanding of why this is going on*, *because…I’m using my earbuds*, *but with the patients*, *I guess the physician’s voice can be heard all over the house*. *So*, *if a husband or somebody else has a comment*, *they actually chime in without even being asked*. *So*, *you get those type of perspectives*.

#### 1. A reassuring alternative to in-person care

Across multiple interviews, physicians agreed that video visits were advantageous because patients were reassured that they could still receive care from a distance and not risk becoming infected with the coronavirus. For instance, one physician noted:

*The fear of coming to the office right now is probably great for many*, *many people*. *And for my patients who are definitely high risk*, *I don’t want them in the office if they don’t need to be there*. *So that I think*, *is a great benefit for the elderly or the immunocompromised*.

Another physician shared a similar view by talking about how their patients appreciated the option of not having in-person visits because it protected them from potential exposure to COVID-19:

*I think having multiple modalities to get in touch with patients has been*, *to me*, *a good thing for the patients in that they feel better about*, *‘Oh*, *I don’t have to come into the clinic and expose myself to COVID*.*’*

#### 2. Convenience of video visits for patients

Several physicians indicated that patients appeared to benefit from remote care delivery because it removed specific barriers to keeping an in-person visit. Specifically, physicians explained how video visits allowed many patients to overcome challenges related to making travel or other personal arrangements for in-person care. For example, patients who typically must take time off to go see their physicians in-person could now complete a video visit while staying at their place of work. As one physician reflected:

*I find that it is really interesting*. *Like sometimes they leave out of their job*, *go to the car*, *do their visit*, *and go back to their job*. *How great is that*? *That took them 20 minutes versus they would have had to leave work at their time*, *I would probably be late*, *they would stay long*, *they would miss half day of work or whatever…So*, *I think it’s actually helped patients to be able to stay at work and do their activities*.

The convenience of remote care delivery also enabled patients greater access to physicians during the early months of the COVID-19 pandemic when stay-at-home orders were in effect: *“I think that it’s good for the fact that people can have access to care*. *I think it’s convenient for patients*.*”*

#### 3. Comfort of receiving care at home

In the same vein, interviewees shared their perspectives about the types of patients who might welcome and potentially benefit from receiving care via telehealth. For example, patients with mental health conditions (e.g., anxiety and depression) and other disabilities could receive primary care from home, where they would face fewer barriers than if they had to engage with PCPs in-person. One PCP explained,

*[Like]for anxious patients*, *they don’t like to drive and see me in clinic*, *right*? *For depressed patients*, *they don’t have the stamina to drag themselves out*. *For people who have*, *[who] are wheelchair dependent or have language barriers*, *or [are] recently a [post-operation patient]*, *they definitely would welcome it*.

Another physician recognized that seeing a patient in their home setting may have the potential to make patients feel more at ease to discuss sensitive health topics, and create a more comfortable environment for patients to engage with their PCP:

*I can’t even believe how much of a barrier that is directed by having people come to you and be in this kind of sterile environment where you have all the control as the physician…*.*[My] perception has been that this is so much more empowering for patients and they’re much more comfortable*, *kind of just sharing and talking about stuff*.

#### 4. Enhanced patient- and family-centered care

Physicians also described how video visits allowed them to get a broader understanding of their patients’ needs because video visits gave them an opportunity to evaluate patients’ homes and gave family members opportunities to engage in the patient’s care. Physicians were “very surprised” to see and meet patients and their families where they were, and this reportedly had the added benefit of providing information about a person’s lifestyle or behaviors that may not have been addressed during an in-person visit:

*Visually seeing where the patient lives has been interesting*. *There are patients who I have seen*, *and then when I see their house*, *I’m very surprised*. *I have met a lot of my patients’ dogs*, *seen a lot of their gardens*, *and it has given me a better understanding of them outside of the four walls of the office*. *So*, *there have been some significant benefits from the ability to actually understand the patient*, *how the patient lives*, *and what they’re doing*. *They also introduce you to their family*, *their caregivers*. *There’s a lot of benefits from that*.

Video visits also helped physicians give patients immediate feedback and instructions about what changes they should make to improve their health. By having access to the patient’s home environment, physicians were able to see what modifications could be made (e.g., discontinue taking a specific medication). One physician explained:

*[Telehealth is] presenting this other window into people’s lives*, *which I think has been curious*. *But it also is good because when I’m like*, *‘Go to the bathroom and get your medication bottles and just show them to me*,*’ [I can say]*, *‘Okay*, *don’t take that one*.*’*

### Physicians’ perspectives about the advantages of remote care delivery in primary care

Across interviews, PCPs also described how the broader transition from in-person visits to remote care delivery during the COVID-19 pandemic had benefited them. Participants noted three main advantages: (1) telehealth accommodated working from home, (2) they received equitable reimbursement for telehealth, and that (3) telehealth promoted work-life balance. Below we describe physicians’ perspectives about these benefits in greater detail, with additional example quotations presented in Table 2.

**Table 2:** Advantages of telehealth for primary care physicians.

Themes	Verbatim comments from primary care physicians
**Accommodating work from home**	*I get to work from home*, *so I can roll out of bed and pretty easily get to work on time…I can go out to my backyard and have lunch*, *and at the end of my day*, *my commute is just a walk back upstairs*. *So*, *from a personal standpoint…it’s just an easy way to do work*.
	*It’s allowed me to see patients on the fly*. *So occasionally*, *somebody would send me a MyChart message or call after hours*.
**Reimbursement for telehealth**	*A lot of providers feel that they’re actually not being paid for the work they do*…*We’re giving them free health care a lot of times*. *We don’t bill for telephone services*. *But now with everything the way it is*, *we bill for telephone services*, *we bill for video visits*. *And so*, *I think from that standpoint*, *it’s just changed the way we think about how we can take care of our patients*. *And it has improved some things*.
	*So*, *if I wanted to do a video visit*, *that’s great*, *I could do a video for a patient*, *but I couldn’t bill for it…[Telehealth] acknowledges a lot of the unreimbursed work that we were doing before*.
**Promotes work-life balance**	*I think having the ability to do telehealth and realizing that you’re not necessarily grinding in the office from 7 a*.*m*. *till 5 p*.*m*. *every day is a little bit enticing [to] people*. *You know*, *being able to do some work from home*, *some in the office; I think it’s a good mix*.
	*Let’s say I’m done [with telehealth visits] at 12 [p*.*m*.*] I’m going to get lunch…I actually have a jump rope and I would jump rope*, *or I would do calisthenics or something like that*. *I can’t do that in the office because I have paperwork to deal with*.

#### 1. Accommodating work-from-home

Physicians noted that providing primary care via telehealth was beneficial because it was convenient. Interviewees provided several examples of why this was advantageous from their perspective, including not having to commute to work. One physician explained how video visits were convenient because they did not have to sacrifice the quality of the care that they provided:

*I mean let’s be honest*, *me being able to provide care from home and I don’t have to leave*, *I don’t have to go anywhere*, *I think it is great*. *I think that’s a great scenario*. *I mean*, *there’s no reason for me to leave the house*. *I’m getting the same interaction as I would if I were in the office*. *I think it’s very convenient*.

Another physician explained how, especially at the beginning of the pandemic, conducting video visits from home mitigated the need to make arrangements for childcare: *“I think it’s created maybe a little bit more flexibility for some of our docs who*, *say they have childcare issues and they can’t come in*, *but they could…still do some visits from home*.*”*

#### 2. Reimbursement for telehealth

Interviewees also indicated that getting reimbursed for virtual visits that were previously unbillable was a significant benefit. As one provider explained, *“Now we’re able to bill for telephone calls and video visits*. *And sometimes those things are occurring kind of on the fly after hours or on weekends*. *And now we have the ability to at least be compensated for that extra work that we’re doing*.*”* Receiving this reimbursement was beneficial, especially in the context of providing video visits to patients who lived further away from the clinic. One physician described how reimbursement parity had opened “the floodgates” and allowed them to provide remote care, “*I do have patients who have traditionally driven far distances*. *And I never did telehealth before because insurance never paid for it*. *So now that the floodgates are so-called open in terms of insurance payment*, *[it] allows me to take care of my patients in the telehealth setting*.*”*

#### 3. Promoting physician work-life balance

Another advantage of telehealth noted by some PCPs was that it enabled physicians to spend more time with their families. One physician reflected, *“I get to eat lunch with my family*. *So*, *the family time actually has improved*. *So*, *if you have kids*, *they don’t think that you’re an absentee parent*, *which I am most of the time*. *I appreciate it*.*”* Additional benefits such as taking breaks in between video visits were reportedly appreciated because they appeared to also impact physician well-being. As one physician explained:

*For me*, *having young children*, *I feel like work-life is not ever balanced*. *But I do feel that doing telehealth and being able to work from home…it help…I get on a telehealth…and eat lunch and I do telehealth*. *And then I finish early with a patient*, *I can go to the bathroom in my own house or go down and get a drink of water or get a snack*, *or something like that; or see the kids*. *So that’s been good*.

## Discussion

Our data provides preliminary evidence that PCPs had positive experiences with delivering remote care during the early months of the COVID-19 pandemic. From the perspective of these physicians, telehealth was able to address patient barriers to in-person care, some of which have been obstacles since before the pandemic (e.g., transportation). In addition, telehealth offered reassurance to patients: providing peace of mind to patients who feared that they would be at-risk for contracting COVID-19 by going to an in-person visit. Further, physicians believed that telehealth created an opportunity to gain a better understanding of patients in their home environments while allowing for family members to play an active role in the patient’s virtual visit. The transition from in-person care to remote care during the COVID-19 pandemic was also reportedly beneficial for PCPs to the extent that it allowed physicians to provide primary care services predominantly from their homes, enabling them to strike a better balance between their work and personal lives.

Our findings complement pre-COVID-19 research that already indicated telehealth is a convenient and an acceptable modality to receive care. A survey-based study found that 87% of patients agreed that video visits with existing providers were more convenient than other ways of getting care, with 93% agreeing that video visits adequately addressed their needs [24]. Our data show similar views during a time of rapid transition from in-person to remote care with the emergence of COVID-19. PCPs in our study reported that patients appeared to appreciate the use of telehealth as an alternative to in-person visits because it offered a comfortable setting in which patients could communicate with their physician [7, 25, 26]. In a similar vein, our findings are aligned with previous research that suggests that telehealth could potentially improve access to care for vulnerable patient populations [27]. While some patient populations, including individuals with health-related social needs [28] and chronic health conditions [29] have been found to be particularly susceptible to missing or cancelling in-person visits, the PCPs in our study suggested that remote care delivery may help alleviate barriers to care for some of these patients, making it possible for them to see a provider without risking their health and safety.

Providers in our study also reportedly appreciated how telehealth offered opportunities to observe patients’ home environments and enabled family members to participate during virtual visits, further highlighting opportunities to design and implement telehealth strategies to enhance patient-centeredness beyond the COVID-19 pandemic. For example, during in-person visits where the use of electronic health records places a focus on completing predetermined tasks and clinical documentation, opportunities for providers to develop a personal understanding of the patient may be curtailed due to the limited time available. Previous studies have found that telehealth has the potential to enhance providers’ understanding of the patient [30, 31], which suggests that remote care delivery could, in practice, improve the delivery of patient-centered care and help patients and providers make more informed decisions based on the needs, goals, and expectations of patients and their families [32]. In contrast, when care is delivered in person, there may be a need to gather and incorporate more personal information into these visits (e.g., a person’s current living conditions, family support needs, and personal preferences for using technology to monitor vital signs) to ensure providers’ understanding of their patients and their preferences. Also, unlike previous reports that say family members only participate in hospital care [33], we found that families can play a role in the delivery of telehealth that is potentially important. More research is warranted to better understand how remote care delivery can both welcome and respect the viewpoints of caregivers and other family members [33, 34] to enhance patient-centered care.

Our findings also complement results of recent quantitative and qualitative studies reporting physicians’ positive attitudes regarding the adoption of telehealth during the COVID-19 pandemic [12, 35]. In our study, which focused on the experiences of PCPs during and shortly after stay-at-home orders had been lifted, physicians acknowledged the benefits of having the increased flexibility to conduct virtual visits from home. This increased flexibility, owing to reimbursement parity for telehealth and state-level policies limiting in-person gatherings, reportedly made it possible for physicians to spend more personal time with their families while also balancing the work demands of standing up a telehealth practice. This is important from a provider perspective because having the ability to schedule remote patient visits conducted from their homes could be one strategy to free up physician time for recovery activities (e.g., sleep, personal time with family members, hobbies) that may be critical to reduce burnout and enhance physician well-being [36]. Much of current published research on clinicians’ perspectives about telehealth only includes data from the beginning of the COVID-19 pandemic [17, 19]. As such, there is a need for further studies to examine how PCPs’ perspectives about telehealth use may have changed over time, especially as primary care delivery has returned to in-person or transitioned to a hybrid model.

Finally, whether and how physicians and practices can sustain the use of remote care delivery strategies into the future deserves further investigation because other factors such as patient barriers to telehealth [37] and reimbursement concerns [27] are likely to influence the accessibility of telehealth moving forward [17, 38, 39]. More research will be necessary to understand how remote care delivery options will impact physician workflows and relationships in their practice settings. For instance, under what circumstances will physicians and support staff make accommodations for patients who prefer remote care to an in-person visit? What type of chronic disease management tasks are more amenable to video visits, and which are not? These and similar types of questions about the use of telehealth [40, 41] will need additional study as we look to the future of remote care delivery.

### Limitations

Our study has several limitations. First, interviews were conducted at a time when physicians were transitioning back from providing only remote care to also delivering in-person care (i.e., June-July 2020). Given that we asked physicians about their experiences using telehealth since the beginning of the COVID-19 pandemic starting in March 2020, our findings are potentially limited by participants’ recall of events that took place during the early phases of the pandemic. Second, we did not interview patients for our study and thus our findings are limited to what physicians reported to be the advantages of delivering remote care for their patient populations. However, given the consistency of responses across interviewees about the themes we present, we are confident that our findings adequately characterize the advantages of telehealth use for physicians as well as their perspectives about benefits for their patients during the early phase of the COVID-19 pandemic. Third, we only recruited PCPs for our study and did not consider the experiences of specialty care providers, whose experiences and perspectives may have been different. Finally, we did not provide a definition of “patient-centered care” to interviewees as we were interested in their own interpretations about the impact of telehealth on patient-centered care; prompting interviewees with a formal definition may have led to different responses given different phrasing of the question.

## Conclusions

In our study of physicians’ perceptions about the increased use of remote care delivery in response to the emergence of COVID-19, interviewees described benefits for both themselves and their patients. Specifically, physicians noted telehealth as a convenient alternative to in-person visits which was further supported by reimbursement parity during the COVID-19 pandemic. Physicians also identified opportunities by which telehealth could enhance the delivery of patient-centered care by offering a glimpse into the home environments of patients and facilitating family engagement during primary care visits. Looking ahead, it is likely that telehealth will remain an important tool to enable remote care for patients, making our improved understanding of this care delivery modality essential.

## Supporting information

S1 File Semi-structured interview guide. (PDF)
