# Supplementary material for: Doctoring from home: Physicians’ perspectives on the advantages of remote care delivery during the COVID-19 pandemic
Source: PLoS One. 2022 Jun 2;17(6):e0269264. doi: 10.1371/journal.pone.0269264 (PMC9162302; doi:10.1371/journal.pone.0269264)
Supplement: S1 File — (PDF) [file pone.0269264.s001.pdf]

Early Impacts of COVID-19 on Primary Care Physicians

<u>Physician Interview Guide</u>

BACKGROUND

First, let us THANK YOU for agreeing to participate in our research project. We are (NAMES) and we are researchers from the College of Medicine at The Ohio State University. We have reached out to you as part of a project we are doing to study how COVID-19 has impacted primary care providers.

As part of this study, we are interested in collecting information from primary care providers about how COVID-19 has impacted their work and ways of working. You have been identified as someone who would provide invaluable information about this topic. We anticipate that this interview will take about 20-30 minutes.

OVERVIEW OF INTERVIEW TOPICS

In this interview, I will ask you a series of open-ended questions to get your perspectives about several topics. These topics include:

- Section 1: Background
- Section 2: Thoughts about changes in your work as a primary care provider
- Section 3: Perspectives about confidence, competence, need for new training
- Section 4: Thoughts about moving to more telehealth, remote care, virtual care options

INTRODUCTION TO INTERVIEW

Before we begin the discussion, we need to take you through an informed consent process. In particular, let me make sure that you understand that:

a. Your participation is completely voluntary. If you do choose to talk with us, you may decide to end the conversation at any time.

b. We consider this discussion to be confidential. Your participation is confidential in the sense that your name will not be used in any reports or articles.

c. We would also like to record the session for the purposes of data collection for our research. The recording will not be used to identify you in any way.

Do you have any questions about our study or this interview process?

PROVIDER INTERVIEW QUESTIONS

Section 1: Background

1. To start, can you please tell me how long you have been a primary care provider (PCP)?
2. What is the name of the organization and the practice where you typically work?
3. What is your regular role in your organization and how long have you been in this role?
4. How many days or sessions per week do you see patients?

Section 2: Thoughts about changes in your work as a primary care provider

5. How has the nature of your work as a PCP changed since COVID-19? (Could you provide us with examples of how your work has changed?)
    - How have any changes brought about by COVID-19 affected how you work with your "usual" support staff?
    - What has helped you in adapting to the changes brought by COVID-19?
6. How do you think primary care in general is being impacted by COVID-19?
7. What challenges have you faced with your current day-to-day work since COVID-19 emerged?
8. What have you found to be positive aspects of your work or "silver linings" since the emergence of COVID-19?
9. What impact do you think COVID-19 will have on how you deliver primary care in the future?

Section 3: Perspectives about confidence, competence, need for new training

10. How have any changes in your work affected your confidence as a provider? (Has anything made you question your abilities as a physician?)
11. If you think about your professional competencies, are there any that you think need to be enhanced since the emergence of COVID-19? (e.g., management skills, communication with patients, technology skills)
12. Have you received new training or retraining since the emergence of COVID-19 to help you with telehealth options such as telemedicine, virtual care, etc.? (Please describe.)
    - What training do you think has been most effective?
    - Is there additional training you wish you had received?
13. Aside from training, are there other resources that would help you deliver care via telehealth?

Section 4: Thoughts about moving to telehealth, remote care, virtual care options

14. How has the shift towards telemedicine (or other virtual care formats) affected your relationships with your patients? (Please describe.)
    - How do you think telemedicine and virtual care options have influenced “patient-centeredness”?
15. How do you feel about delivering care via telemedicine or other virtual care modalities? (Please describe.)
16. Do you have any concerns about the role of telemedicine and virtual primary care in the future?
17. What feedback, if any, have you received from patients about telehealth or remote care options?
18. What challenges have you perceived patients to experience with telehealth?
19. What do you think can help address these challenges?

INTERVIEW CLOSURE AND FOLLOW UP

20. Is there anything else you would like to share about how your work has changed since the emergence of COVID-19?

THANK YOU

Thank you so much for your time and participation. Your participation was extremely helpful.
